# Hierarchical 0D−2D Co/Mo Selenides as Superior Bifunctional Electrocatalysts for Overall Water Splitting

**DOI:** 10.3389/fchem.2020.00382

**Published:** 2020-05-19

**Authors:** Lu Xia, Hao Song, Xingxing Li, Xuming Zhang, Biao Gao, Yang Zheng, Kaifu Huo, Paul K. Chu

**Affiliations:** ^1^The State Key Laboratory of Refractories and Metallurgy and Institute of Advanced Materials and Nanotechnology, Wuhan University of Science and Technology, Wuhan, China; ^2^The College of Resources and Environment Engineering, Wuhan University of Science and Technology, Wuhan, China; ^3^Departments of Physics, Materials Science and Engineering, and Biomedical Engineering, City University of Hong Kong, Hong Kong, China

**Keywords:** cobalt selenide, molybdenum selenide, hierarchical heterostructure, bifunctional electrocatalysts, overall water splitting

## Abstract

Development of efficient electrocatalysts combining the features of low cost and high performance for both the hydrogen evolution reaction (HER) and oxygen evolution reaction (OER) still remains a critical challenge. Here, we proposed a facile strategy to construct *in situ* a novel hierarchical heterostructure composed of 0D−2D CoSe_2_/MoSe_2_ by the selenization of CoMoO_4_ nanosheets grafted on a carbon cloth (CC). In such integrated structure, CoSe_2_ nanoparticles dispersed well and tightly bonded with MoSe_2_ nanosheets, which can not only enhance kinetics due to the synergetic effects, thus promoting the electrocatalytic activity, but also effectively improve the structural stability. Benefiting from its unique architecture, the designed CoSe_2_/MoSe_2_ catalyst exhibits superior OER and HER performance. Specifically, a small overpotential of 280 mV is acquired at a current density of 10 mA·cm^−2^ for OER with a small Tafel slope of 86.8 mV·dec^−1^, and the overpotential is 90 mV at a current density of 10 mA·cm^−2^ for HER with a Tafel slope of 84.8 mV·dec^−1^ in 1 M KOH. Furthermore, the symmetrical electrolyzer assembled with the CoSe_2_/MoSe_2_ catalysts depicts a small cell voltage of 1.63 V at 10 mA·cm^−2^ toward overall water splitting.

## Introduction

Hydrogen is a promising energy source that boasts a high power density and environmental friendliness; therefore, electrolysis of water is hotly pursued as a renewable, efficient, and pollution-free technique (Amiinu et al., 2017; Luo et al., 2018; Zhu et al., 2018). Electrocatalytic water splitting consists of the hydrogen evolution reaction (HER) and oxygen evolution reaction (OER), and electrocatalysts as the chemical reaction centers play a critical role in the water splitting electrolyzer. Although some noble metal oxide catalysts (RuO_2_ and IrO_2_) have high electrocatalytic performance for the OER and some noble metal catalysts (Pt and Ir) deliver good electrochemical property in the HER, the high cost and scarcity restrict their wide industrial application (Trasatti, 1972, 1984). Therefore, noble-metal-free catalysts with high stability and efficiency are crucial to large-scale hydrogen production from water splitting. Currently, the OER activity in alkaline solution is the bottleneck in overall water splitting due to the sluggish kinetics arising from the multiproton-coupled electron transfer steps (Jamesh and Sun, 2018). In practice, the HER catalyst in the electrolyzer should be compatible with the OER catalyst and functions in the same medium. Hence, development of suitable bifunctional noble-metal-free electrocatalyts with both high HER and OER performance in alkaline media is of great significance.

In recent years, transition metal dichalcogenides (TMDs) have attracted significant research interests owing to their earth-abundant reserves and acceptable activity for electrocatalytic HER (Xie et al., 2013; Zhang et al., 2017a; Xue et al., 2018; Wang et al., 2019). Particularly, layered MoSe_2_ has been considered as a promising HER electrocatalyst because of its unique structure features and high electrochemical activity (Shi et al., 2015; Chen et al., 2018a; Zhang et al., 2018). Theoretical research has demonstrated that the Gibbs free energy for H atom absorption on the edge of MoSe_2_ is lower than that of MoS_2_ due to the more metallic nature of MoSe_2_, revealing the higher HER performance (Tang et al., 2014; Lai et al., 2017; Yang et al., 2018). In addition, it also has been experimentally confirmed that the unsaturated Se edges in MoSe_2_ nanosheets are extremely active as the S edges in MoS_2_, which is responsible for the high HER activity (Jaramillo et al., 2007; Tang and Jiang, 2016). However, similar to MoS_2_, the HER activity of layered MoSe_2_ is largely limited by its poor conductivity and serious aggregation or restacking during the synthesis procedure (Mao et al., 2015; Qu et al., 2015), inhibiting the practical application of MoSe_2_ catalyst. Therefore, it is significant to improve the electrochemical activity of MoSe_2_-based catalyst. Recent works have shown that coupling MoSe_2_ with other transition metal selenides and constructing heterostrucurted materials could be an effective approach to further enhance the electrochemical performance of MoSe_2_. For instance, Wang et al. found that the MoSe_2_@Ni_0.85_Se nanowire delivered enhanced kinetics and performance for HER in alkaline conditions due to the high density of active edges of MoSe_2_ and the good conductivity of Ni_0.85_Se (Wang et al., 2017a). Zhang et al. synthesized 3D MoSe_2_/NiSe_2_ nanowires, which significantly enhanced HER activity with a low Tafel slope and overpotential in 0.5 M H_2_SO_4_, because the 3D structure affords more active sites (Zhang et al., 2017a). Liu et al. fabricated MoSe_2_-NiSe@carbon heteronanostructures and achieved glorious HER catalytic properties and excellent durability in both acidic and base conditions (Liu et al., 2018). In addition, the hierarchical mesoporous MoSe_2_@CoSe/N–C composite also exhibits outstanding HER activity (Chen et al., 2019b). Despite significant success, most of previous reports mainly focused on the improvement of HER performance, while the OER activity of MoSe_2_ catalyst in alkaline media has been ignored. Hence, the rational design and fabrication of MoSe_2_-based bifunctional electrocatalysts with satisfactory activity and stability toward overall water splitting in alkaline solution still remain a big challenge.

**Figure 1:**
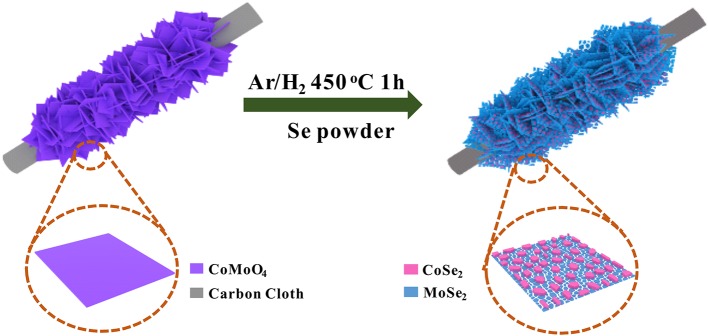
Schematic illustration of the fabrication of CoSe_2_/MoSe_2_.

In this work, we developed a facile *in situ* phase separation strategy to construct a novel hierarchical heterostructure consisting of 0D−2D CoSe_2_/MoSe_2_ via the selenization of CoMoO_4_ nanosheets supported on a carbon cloth (CC) (Figure 1). Due to the *in situ* phase transformation, CoSe_2_ nanoparticles are uniformly anchored on MoSe_2_ nanosheets in the integrated structure, which can not only enhance reaction kinetics because of the synergetic effects, thus boosting the electrocatalytic activity, but also effectively suppress the aggregation/restacking of MoSe_2_ nanosheets, thereby improving the structural stability. Moreover, the hierarchical structure assembled by 0D−2D CoSe_2_/MoSe_2_ could provide abundant active sites for the electrochemical reactions. As a result, the designed CoSe_2_/MoSe_2_ architecture exhibits outstanding OER and HER performance in alkaline media. More specifically, a small overpotential of 280 mV is achieved at a current density of 10 mA·cm^−2^ for OER with a small Tafel slope of 86.8 mV·dec^−1^, and the overpotential is 90 mV at a current density of 10 mA·cm^−2^ for HER with a Tafel slope of 84.8 mV·dec^−1^ in 1 M KOH. Moreover, the symmetrical electrolyzer assembled with the CoSe_2_/MoSe_2_ catalysts delivers a small cell voltage of 1.63 V at 10 mA·cm^−2^ toward overall water splitting.

## Experimental Section

### Synthesis of CoMoO_4_ Nanosheet

Firstly, a pristine carbon cloth (CC) was treated with nitric acid solution overnight, subsequently ultrasonicated in deionized (DI) water and dried in an oven at 80°C for 2 h. After that, 1 mmol cobalt acetate, 1 mmol ammonium molybdate, 2 mmol urea, and 5 mmol ammonium fluoride were dissolved in 30 mL DI water, followed by ultrasonication for 30 min. Then, the homogeneous solution was poured into a 50-mL Teflon-lined stainless autoclave with the CC kept at 150°C for 6 h. After cooling to room temperature, the CC was washed with DI water for several times and dried in a vacuum freeze-dryer overnight. Finally, the obtained sample was treated at 400°C for 2 h with a ramp rate of 5°C min^−1^ in an argon atmosphere.

### Synthesis of CoSe_2_/MoSe_2_

The as-prepared CoMoO_4_ precursor was reacted with 0.5 g selenium powder at 450°C for 1 h under an Ar/H_2_ (90%/10%) atmosphere to form the CoSe_2_/MoSe_2_.

### Synthesis of CoSe_2_

The CoSe_2_ was prepared through two steps. Firstly, the treated CC was immersed in a 0.1 M Co(NO_3_)_2_ solution for the electrodeposition of Co (Yang et al., 2015). Then, the collected sample was reacted with 0.5 g selenium powder under an Ar/H_2_ (90%/10%) atmosphere at 450°C for 1 h.

### Synthesis of MoSe_2_

Firstly, MoS_2_ was prepared via hydrothermal reaction with the CC at 200°C for 12 h, followed by heating at 400°C for 2 h to form MoO_3_ (Wu et al., 2018). Then, the obtained MoO_3_ was reacted with 0.5 g selenium powder at 450°C for 1 h under an Ar/H_2_ (90%/10%) atmosphere.

### Preparation of Pt/C

Four milligrams of 20% Pt/C and 20 μL 5% Nafion solution were added into 1 mL solution of isopropanol and DI water (9:1) and then sonicated to form a uniform solution. Finally, the 1^*^1 cm^2^ CC was soaked in the homogeneous solution and dried in air at atmospheric temperature.

### Preparation of RuO_2_

Four milligrams of RuO_2_ and 20 μL 5% Nafion solution were added into 1 mL solution of isopropanol and DI water (9:1), and then the sample was sonicated to form a uniform solution. Finally, the 1^*^1 cm^2^ CC was soaked in the homogeneous solution and dried in air at atmospheric temperature.

## Characterization

The phase composition of the samples were characterized by X-ray diffraction (XRD, Bruker D8A A25), and the chemical states were determined through X-ray photoelectron spectroscopy (XPS, ESCALB 250Xi). The morphology and microstructure were recorded via field emission scanning electron microscopy (FE-SEM, FEI Nova NANOSEM 400) and high-resolution transmission electron microscopy (HR-TEM, JEM-2100 UHR STEM).

## Electrochemical Measurements

All samples made use of a three-electrode system performed by a biologic VSP300 type electrochemical workstation (Biologic Science Instruments, France). The sample of CoSe_2_/MoSe_2_ was put on the electrode holder as the working electrode with a mass loading of 4 mg/cm^2^, the saturated calomel electrode (SCE) was the reference electrode, and a carbon rod served as the counter electrode. The electrolyte was 1 M KOH solution with saturated N_2_. Linear sweep voltammetry (LSV) was characterized by polarization curves of OER with a scanning rate of 5 mV s^−1^ from 0 to 0.8 V vs. SCE. Similarly, the polarization curves of HER were determined under the same condition from 0 to −0.8 V vs. SCE. The potentials were standardized by a reversible hydrogen electrode (RHE) as shown in the following: E (RHE) = E (SCE) + 0.059 × pH with instrument automatic 85% iR compensation. The electrochemically active surface area (ECSA) was calculated by cyclic voltammetry (CV) performed from −0.3 to −0.2 V vs. SCE with different scanning rates of 40, 60, 80, 100, and 120 mV s^−1^. Electrochemical impedance spectroscopy (EIS) measurements were conducted by biologic VMP3 (Biologic Science Instruments, France) from 100 KHz to 0.1 Hz. The overall water-splitting electrolyzer was performed with CoSe_2_/MoSe_2_ as electrodes and 1 M KOH as the electrolyte.

## Results and Discussion

**Figure 2:**
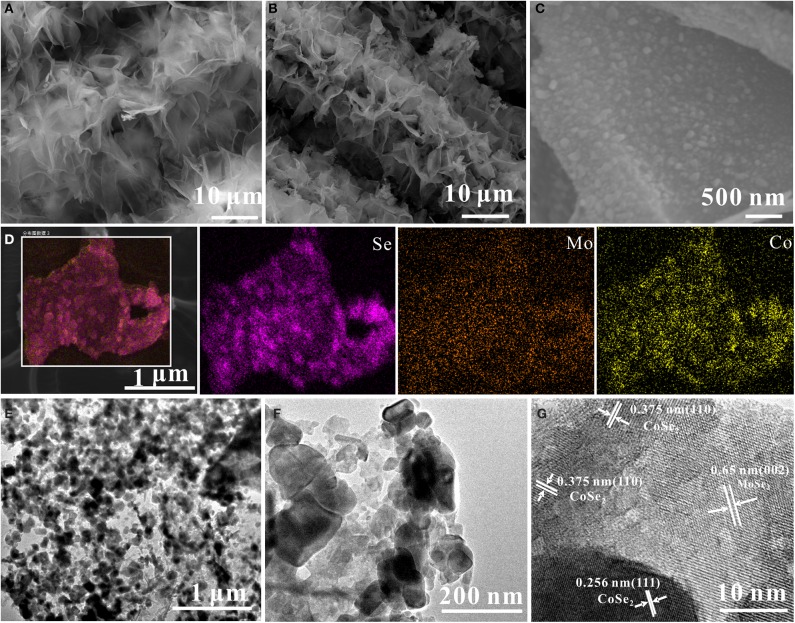
FE-SEM images of **(A)** CoMoO_4_ and **(B,C)** CoSe_2_/MoSe_2_; **(D)** elemental maps of CoSe_2_/MoSe_2_; **(E,F)** TEM of CoSe_2_/MoSe_2_; **(G)** HR-TEM images of CoSe_2_/MoSe_2_.

Figure 2A presents the FE-SEM image of the as-prepared CoMoO_4_ precursor, which presents uniform nanosheets (with a lateral size of 2 μm) perpendicularly grown on the CC substrate with high coverage. After a selenization process, the obtained CoSe_2_/MoSe_2_ sample well maintains the pristine morphology of the CoMoO_4_ precursor (Figure 2B). Moreover, the high-magnification SEM image further reveals that lots of nanoparticles are well dispersed on the surface of the nanosheet (Figure 2C), implying the structure and phase evolution during the selenization treatment. The elemental maps in (Figure 2D) show that Mo, Co, and Se are uniformly distributed throughout the nanosheets. In addition, the low-resolution TEM images in (Figures 2E,F) display that nanoparticles are uniformly distributed on the nanosheet during the thermal reduction procedure, forming the 0D/2D structure. Furthermore, the high-resolution TEM (Figure 2G) shows the lattice fringes of 0.26 nm and 0.65 nm corresponding to the (111) and (002) planes of CoSe_2_ and MoSe_2_, respectively (Qu et al., 2016; Liu et al., 2017), demonstrating the successful formation of the CoSe_2_/MoSe_2_ after the selenization reaction.

**Figure 3:**
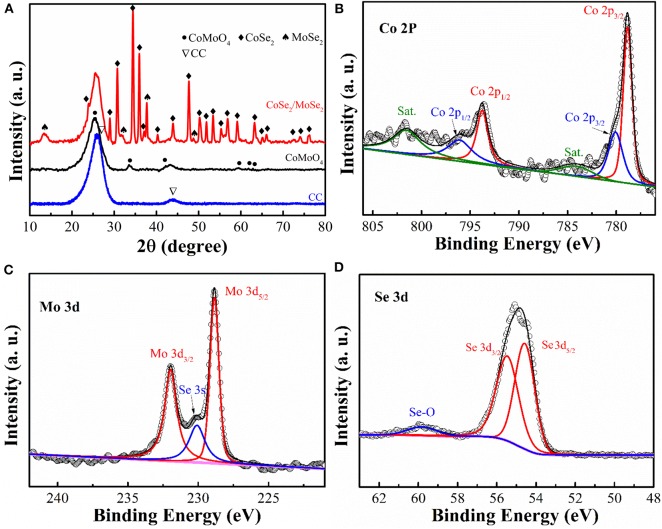
**(A)** XRD patterns of CC, CoMoO_4_, and CoSe_2_/MoSe_2_; high-resolution XPS spectra of **(B)** Mo 3d, **(C)** Co 2p, and **(D)** Se 3d of CoSe_2_/MoSe_2_.

To investigate phase evolution during the selenization process, the crystal structure and phase composition of the obtained samples were characterized by X-ray diffraction (XRD) analysis (Figure 3A). The diffraction peaks of CoMoO_4_ precursor (in the black line) can be well indexed to the CoMoO_4_ phase (JCPDS No: 21-0868) (Wang et al., 2016). After the thermal reduction, some new diffraction peaks can be observed. The diffraction peaks at around 13.7 °, 27.6 °, 31.4 °, and 37.8 ° can be assigned to the MoSe_2_ phase (JCPDS No: 77-1715) (Qu et al., 2016), while the other peaks could be attributed to the phase of CoSe_2_ (JCPDS No:53-0449) (Liu et al., 2017). The XRD result clearly manifests the successful phase separation of the CoSe_2_ and MoSe_2_ from the CoMoO_4_ precursor via the selenization process.

X-ray photoelectron spectroscopy (XPS) measurement was carried out to analyze the composition and chemical state of as-prepared samples. (Figure 3B) illustrates the high-resolution Co 2p peaks at 778.8 eV (Co 2p_3/2_), 793.7 eV (Co 2p_1/2_), 780 eV (Co 2p_3/2_), and 796 eV (Co 2p_1/2_), corresponding to CoSe_2_ and cobalt–oxide bond, while those peaks at 784.1 eV and 801.5 eV are the satellite peaks (Mu et al., 2016; Wang et al., 2017b; Gao et al., 2018). Furthermore, the fine Mo 3d XPS spectrum (Figure 3C) shows the main peaks at 228.8 eV and 231.9 eV, which represent the Mo 3d_5/2_ and Mo 3d_3/2_ of MoSe_2_ (Wang et al., 2018a,b). Additionally, the peak located at 230 eV can be ascribed to the Se 3s of MoSe_2_ (Zhao et al., 2018). The Se 3d XPS spectrum (Figure 3D) displays the characteristic of CoSe_2_ and MoSe_2_ at 54.5 eV and 55.4 eV in agreement with the Se 3d_5/2_ and Se 3d_3/2_, respectively (Gao et al., 2018). Moreover, the peak at around 59.8 eV is confirmed to correspond to the selenium–oxygen bond (Kong et al., 2014). According to these results, the selenization process induced the phase separation from CoMoO_4_ into the nanoscale CoSe_2_ and MoSe_2_.

**Figure 4:**
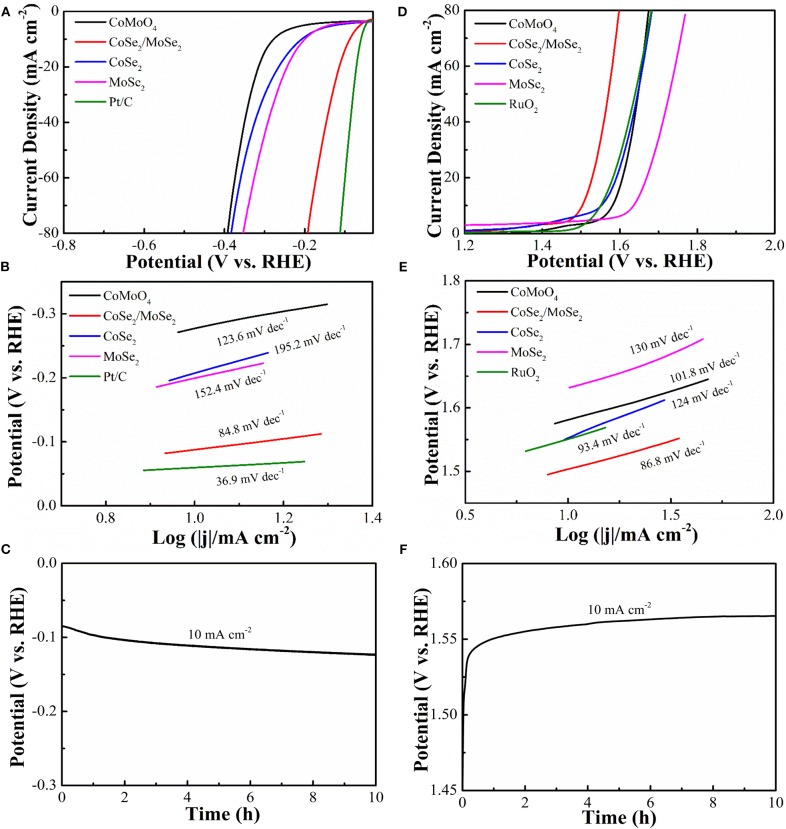
**(A)** HER polarization curves of CoMoO_4_, CoSe_2_/MoSe_2_, CoSe_2_, MoSe_2_, and Pt/C; **(B)** Tafel slopes; **(C)** galvanostatic of CoSe_2_/MoSe_2_ for HER; **(D)** OER polarization curves of CoMoO_4_, CoSe_2_/MoSe_2_, CoSe_2_, MoSe_2_, and RuO_2_; **(E)** Tafel slopes in OER; **(F)** galvanostatic of CoSe_2_/MoSe_2_ for OER.

It is generally recognized that highly efficient electrocatalysts worked in alkaline solution is the bottleneck for large-scale application of overall water splitting. Linear sweep voltammetry (LSV) at a scanning rate of 5 mV s^−1^ was characterized by the electrocatalytic HER and OER capacities of the samples by a three-electrode system in 1 M KOH solution with saturated N_2_. By contrast, CoSe_2_, MoSe_2_ (Figure S1), RuO_2_, and Pt/C catalysts were performed in the same condition. The HER polarization curves and corresponding Tafel slops are depicted in (Figures 4A,B). The overpotential (η_10_) and Tafel slope of CoSe_2_/MoSe_2_ are 90 mV and 84.8 mV·dec^−1^, which are better than those of CoMoO_4_ (277 mV, 123.6 mV·dec^−1^), CoSe_2_ (205 mV, 195.2 mV·dec^−1^), and MoSe_2_ (199 mV, 152.4 mV·dec^−1^). CoSe_2_ has a metallic character, which can promote the dissociation of water and provide protons under alkaline conditions, thus improving the HER performance of MoSe_2_ (Kwak et al., 2016). In addition, the hierarchical nanosheet array assembled by the CoSe_2_/MoSe_2_ provides abundant active sites for the electrochemical reaction at the phase interface, which can further enhance the HER performance (Zhang et al., 2017a). Therefore, the CoSe_2_/MoSe_2_ catalyst exhibits improved HER performance benefiting from the synergistic effect. The catalyst of Pt/C illustrates an overpotential (η_10_) (59 mV) and Tafel slope (36.9 mV·dec^−1^) in 1 M KOH that are similar to those in other literatures (Chen et al., 2018b; Wan et al., 2018). Moreover, the overpotential of CoSe_2_/MoSe_2_ is superior to those of recently reported selenide catalysts such as NiSe NWs/Ni Foam (96 mV) (Tang et al., 2015), EG/cobalt selenide/NiFe–LDH (260 mV) (Hou et al., 2016), o-CoSe_2_/P (104 mV) (Zheng et al., 2018), CoSe_2_ NCs (520 mV) (Kwak et al., 2016), Co_0.75_Ni_0.25_Se/NF (106 mV) (Liu et al., 2019), 1T MoSe_2_/NiSe (120 mV) (Zhang et al., 2019), and SWCNTs/MoSe_2_ (219 mV) (Najafi et al., 2019) (Table S1).

The electrocatalytic OER properties are determined by LSV and polarization measurements as shown in (Figures 4D,E). The CoSe_2_/MoSe_2_ catalyst shows a lower overpotential (η_10_ of 280 mV) than those of the CoMoO_4_ (352 mV), CoSe_2_ (322 mV), MoSe_2_ (404 mV), and RuO_2_ (318 mV), respectively. More importantly, the OER performance of the designed CoSe_2_/MoSe_2_ sample exceeds those of recently reported selenide catalysts in OER, for instance, the Ag–CoSe_2_ (320 mV) (Zhao et al., 2017), CoSe_2_ NCs (430 mV) (Kwak et al., 2016), CoSe_2_/DETA (392 mV) (Guo et al., 2017), NiCo_2_Se_4_ holey nanosheets (295 mV) (Fang et al., 2017), NiSe–Ni_0.85_Se/CP (300 mV) (Chen et al., 2018a), SWCNTs/MoSe_2_ (295 mV) (Najafi et al., 2019), 1T/2H MoSe_2_ (397 mV) (Li et al., 2019), and CoSe_2_@MoSe_2_ (309 mV) (Chen et al., 2019c) (Table S2). Furthermore, the corresponding Tafel slope of CoSe_2_/MoSe_2_ is 86.8 mV·dec^−1^, which is smaller than those of the CoMoO_4_ (101.8 mV·dec^−1^), CoSe_2_ (124 mV·dec^−1^), MoSe_2_ (130 mV·dec^−1^), and RuO_2_ (93.4 mV·dec^−1^). The CoSe_2_/MoSe_2_ has lower overpotential and smaller Tafel, which can be attributed to its unique hierarchical heterostructure, facilitating electron transfer and accelerating OER kinetics. In this heterostructure, the transfer of electrons from CoSe_2_ phase to MoSe_2_ phase in the CoSe_2_/MoSe_2_ interface can result in electron-poor Co species and electron-rich Mo species (Liu et al., 2018). It is believed that the Se anion can affect the electron transfer between Co and Mo species, which is important for boosting catalytic ability (Yan et al., 2019). Besides, the formation of CoOOH is the primary cause to promote OER activity (Liu et al., 2015), and the increased 3d−4p repulsion between the center of the metal d band and the center of the p band of the Se site further promotes the rapid transfer of dioxygen molecules, thus improving OER performance (Li et al., 2017).

To understand the effects of the structure and composition of prepared catalyst on the electrochemical performance, several CoSe_2_/MoSe_2_ catalysts were collected at different selenization temperatures and the HER and OER performance were evaluated by LSV analysis (Figure S2). It can be seen that the CoSe_2_/MoSe_2_ sample obtained at 450°C (CoSe_2_/MoSe_2_-450) possesses better electrocatalytic properties than other counterparts, which can be ascribed to its superior structure. As shown in (Figure S3), with the selenization temperature increasing, the size of nanoparticles on the surface of nanosheets increased as well, indicating higher crystallinity. Generally, larger particle size will reduce the active surface of catalyst (Zhang et al., 2017b; Chen et al., 2019a). Therefore, when the selenization temperature elevated to 500°C (CoSe_2_/MoSe_2_-500), the catalytic performance slightly declined owing to its larger particle size and lower active surface. In addition, (Figure S4) displays the composition of the CoSe_2_/MoSe_2_ catalysts achieved at a different selenization temperature. As can be seen, when the selenization process proceeded at low temperature, the obtained CoSe_2_/MoSe_2_ catalyst has poor MoSe_2_ phase and low crystallinity, which are responsible for the poor electrochemical catalytic performance of the catalysts (CoSe_2_/MoSe_2_-350 and CoSe_2_/MoSe_2_-400). Therefore, the catalyst synthesized at 450°C shows the best performance, benefiting from the appropriate crystal structure and phase composition.

**Figure 5:**
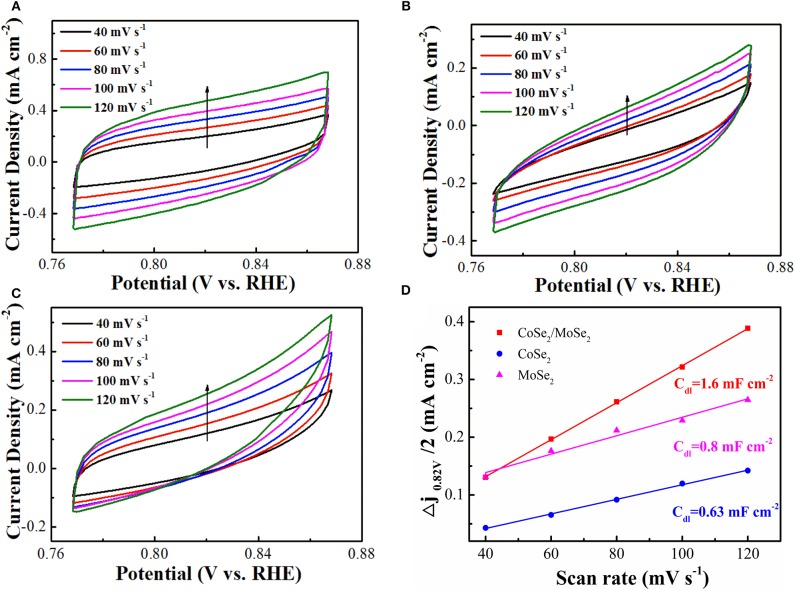
Electrochemical double-layer capacitance with the CV curves acquired at different scanning rates from 40, 60, 80, 100, and 120 mV s^−1^: **(A)** CoSe_2_/MoSe_2_, **(B)** CoSe_2_, and **(C)** MoSe_2_; **(D)** current densities (Δj = janode – jcathode, at 0.82 V) as a function of scanning rates of CoSe_2_/MoSe_2_, CoSe_2_, and MoSe_2_ with the corresponding slope being twice that of the C_dl_ values.

The electrochemically active surface area (ECSA) of as-prepared catalyst was evaluated by the double-layer capacitance (*C*_*dl*_), which was measured by CV in a non-Faradaic reaction potential range (Deng et al., 2015). The *C*_*dl*_ values of the CoSe_2_/MoSe_2_ (1.6 mF cm^−2^) is higher than those of CoSe_2_ (0.63 mF cm^−2^) and MoSe_2_ (0.8 mF cm^−2^), as shown in (Figure 5), suggesting more active sites of the CoSe_2_/MoSe_2_ catalyst. Furthermore, the smaller *R*_*ct*_ value for the CoSe_2_/MoSe_2_ catalyst in the EIS measurement (Figure S5) implies the promoted charge transfer and boosted kinetics, which can be ascribed to the abundant interfaces and synergetic effect between the CoSe_2_ and MoSe_2_.

The structural stability is another significant parameter for catalysts in HER and OER. (Figures 4C,F) show a galvanostatic for CoSe_2_/MoSe_2_ catalyst in both the HER and OER processes. The morphology and composition of the catalyst after galvanostatic cycling are characterized by SEM and XPS. The CoSe_2_/MoSe_2_ could well inherit the pristine sheet-like structure, demonstrating good structural stability. In addition, the fine XPS spectra of the Co 2p, Mo 3d, Se 3d acquired from the sample of CoSe_2_/MoSe_2_ after galvanostatic measurement confirm the reservation of CoSe_2_ and MoSe_2_ (Figure S6), indicating phase stability during the electrochemical reactions.

**Figure 6:**
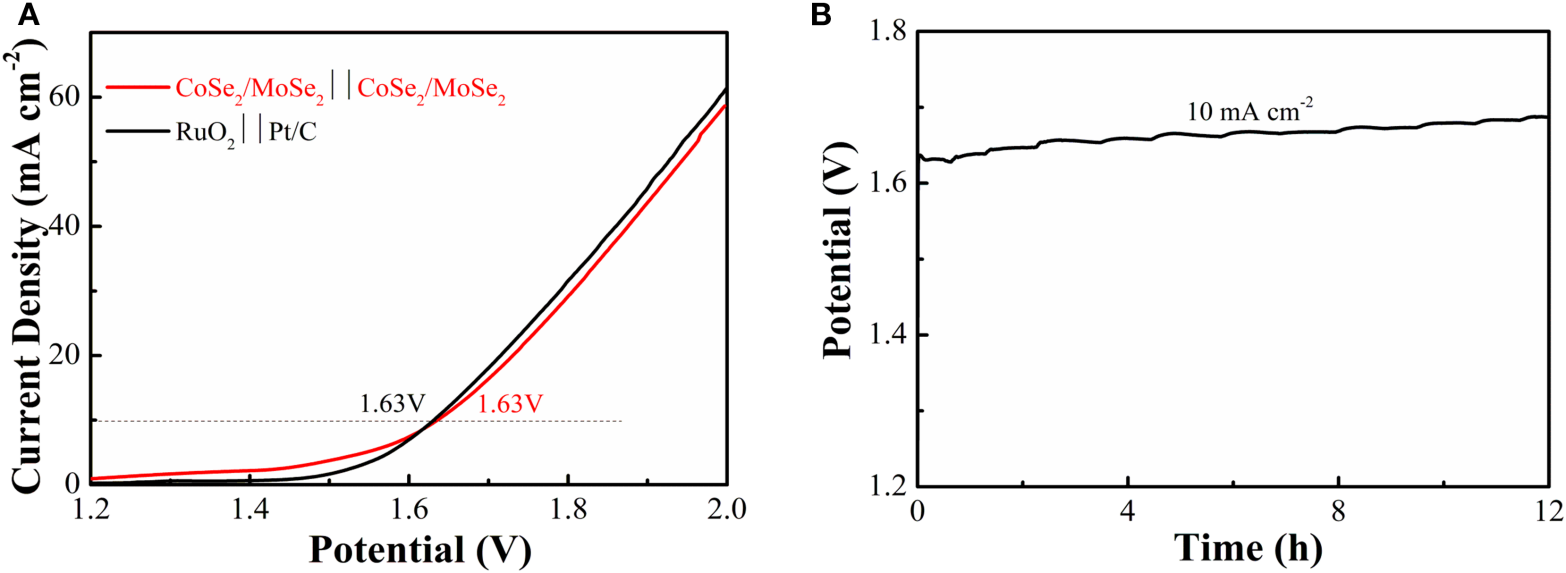
**(A)** LSV curves of water splitting with CoSe_2_/MoSe_2_ as the anode and cathode; **(B)** galvanostatic testing of the CoSe_2_/MoSe_2_-based water splitting electrolyzer for 12 h at 10 mA cm^−2^.

To investigate its practical application of the obtained catalyst, an overall water splitting electrolyzer is assembled with CoSe_2_/MoSe_2_ as electrodes in 1 M KOH. It can decompose water at a low cell voltage of 1.63 V (current density at 10 mA·cm^−2^) (Figure 6A), and the efficiency is similar to those constituting of the noble-metal-based cathode and anode (RuO_2_ vs. Pt/C). Moreover, the overall water splitting performance of the CoSe_2_/MoSe_2_ is better than those of other recently reported non-noble metals at the same current density, such as (Ni,Co)_0.85_Se NSAs (1.65 V) (Xiao et al., 2018), a-CoSe/Ti mesh (1.65 V) (Liu et al., 2015), CoO_x_-CoSe (1.64 V) (Xu et al., 2016), Co_0.85_Se@NC (1.76 V) (Meng et al., 2017), CoB_2_/CoSe_2_ (1.73 V) (Guo et al., 2017), NiSe_2_/Ni (1.64 V) (Zhang et al., 2018), 1T/2H MoSe_2_/MXene (1.64 V) (Li et al., 2019), and Ni_3_Se_2_/CF (1.65 V) (Shi et al., 2015) (Table S3). Additionally, CoSe_2_/MoSe_2_ electrolyzer exhibits a slight increase in the potential after being cycled for 12 h in alkaline solution (Figure 6B).

## Conclusion

In summary, a novel hierarchical 0D−2D Co/Mo selenide was developed by a facile *in situ* phase separation strategy. Benefiting from its unique structure and composition, the constructed CoSe_2_/MoSe_2_ catalyst exhibits small η_10_ of 280 mV and 90 mV and Tafel slopes of 86.8 mV·dec^−1^ and 84.8 mV·dec^−1^ for OER and HER, respectively. Furthermore, the electrolyzer comprising CoSe_2_/MoSe_2_ as the bifunctional catalyst shows a small water splitting cell voltage of 1.63 V at a current density of 10 mA·cm^−2^. This work provides insights into rational design and development of economical and valid bifunctional catalysts for overall water splitting.

## Data Availability Statement

All datasets generated for this study are included in the article/Supplementary Material.

## Author Contributions

LX implemented the experiment, analyzed the data and wrote the article. HS, XL, XZ, and BG participated in the formulation of the experimental scheme. YZ, KH, and PC revised the article.

## Conflict of Interest

The authors declare that the research was conducted in the absence of any commercial or financial relationships that could be construed as a potential conflict of interest.
