# Supplementary material for: Hierarchical 0D−2D Co/Mo Selenides as Superior Bifunctional Electrocatalysts for Overall Water Splitting
Source: Front Chem. 2020 May 19;8:382. doi: 10.3389/fchem.2020.00382 (PMC7248173; doi:10.3389/fchem.2020.00382)
Supplement: Supplementary file 1 [file Table_1.docx]

**Supporting Information:**

**Hierarchical 0D-2D Co/Mo selenides as Superior Bifunctional Electrocatalysts for Overall Water Splitting**

Lu Xia*^1,2^*, Hao Song *^1^*, Xingxing Li *^1^*, Xuming Zhang *^1,^**, Biao Gao *^1, 3^*, Yang Zheng *^1,^** Kaifu Huo *^1^*, Paul K. Chu *^3^*

*1 The State Key Laboratory of Refractories and Metallurgy and Institute of Advanced Materials and Nanotechnology, Wuhan University of Science and Technology, Wuhan 430081, China.*

*2 The College of Resources and Environment Engineering, Wuhan University of Science and Technology, Wuhan 430081, China.*

*3 Department of Physics, Department of Materials Science and Engineering, and Department of Biomedical Engineering, City University of Hong Kong, Tat Chee Avenue, Kowloon, Hong Kong, China)*

^*^Corresponding authors:

E-mail: xumzhang@wust.edu.cn (X. Zhang); yzheng@wust.edu.cn (Y. Zheng)


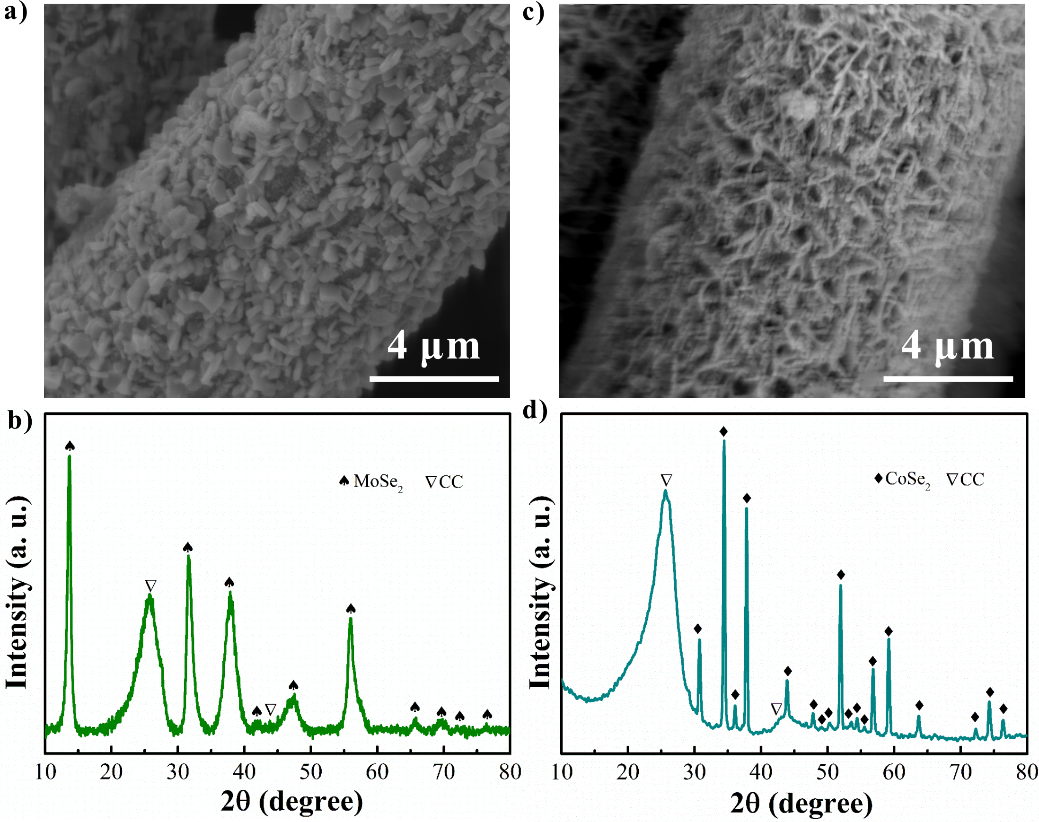


**Figure S1.** FE-SEM images of (a) MoSe_2_, (c) CoSe_2_; XRD patterns of (b) MoSe_2_, (d) CoSe_2._


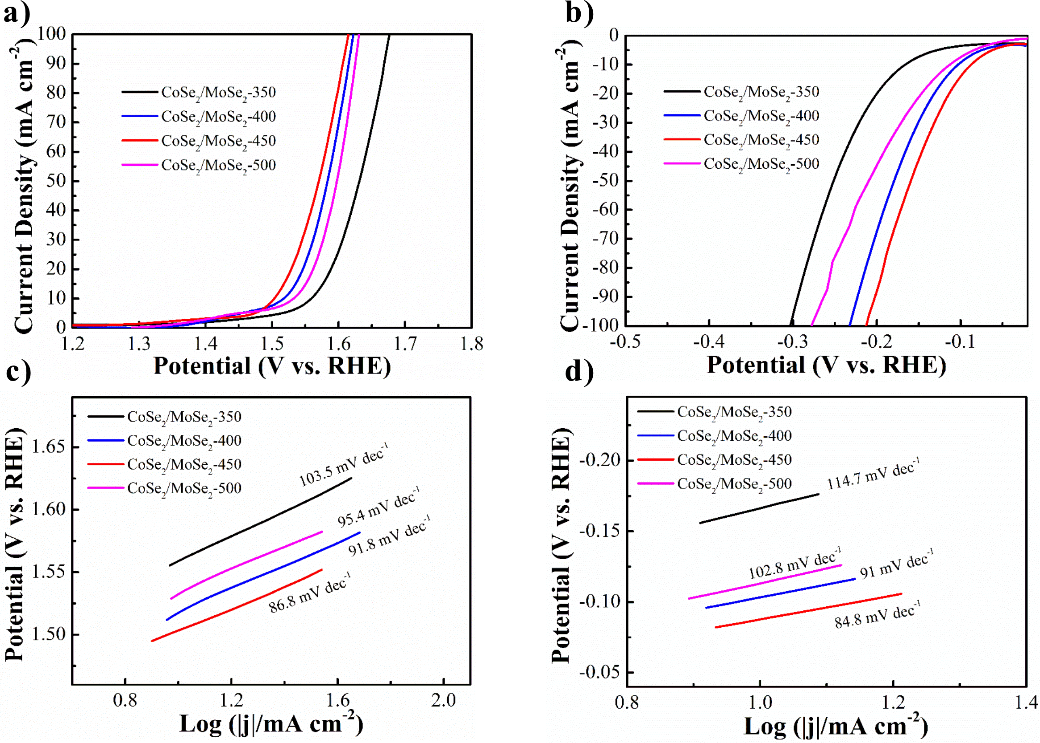


**Figure S2.** **(**a) OER polarization curves of CoSe_2_/MoSe_2_ with different temperatures; (b) HER polarization curves of CoSe_2_/MoSe_2_ with different temperatures; (c) Tafel slopes in OER; (d) Tafel slopes in HER.


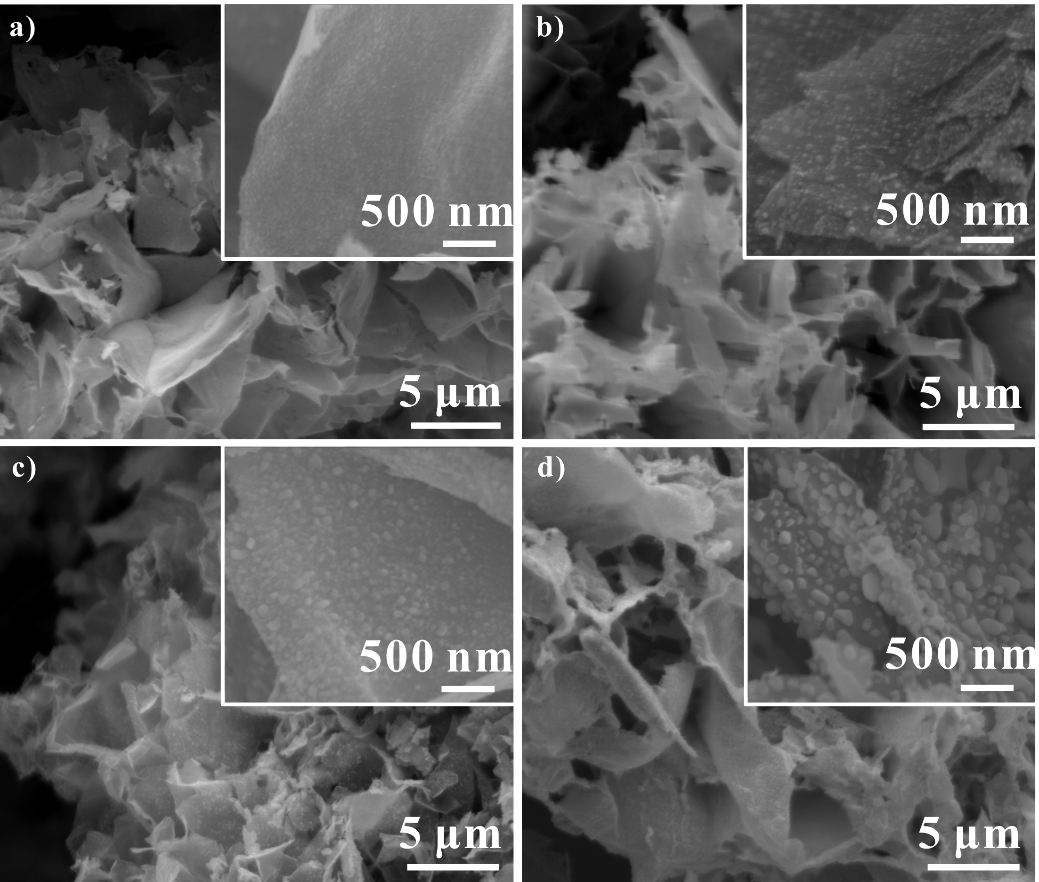


**Figure S3.** FE-SEM images of CoSe_2_/MoSe_2_ for different selenization temperatures. (a) 350 ^o^C,(b) 400 ^o^C,(c) 450 ^o^C,(d) 500 ^o^C.


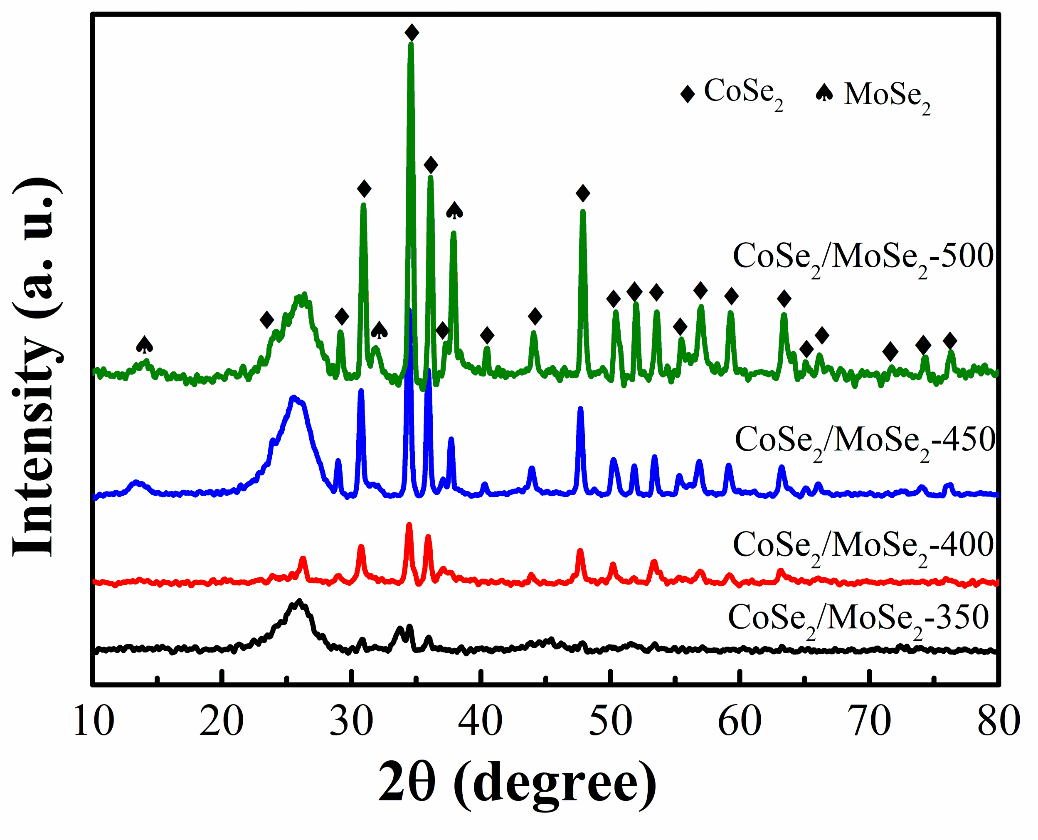


**Figure S4.** XRD patterns of CoSe_2_/MoSe_2_ with different temperatures.


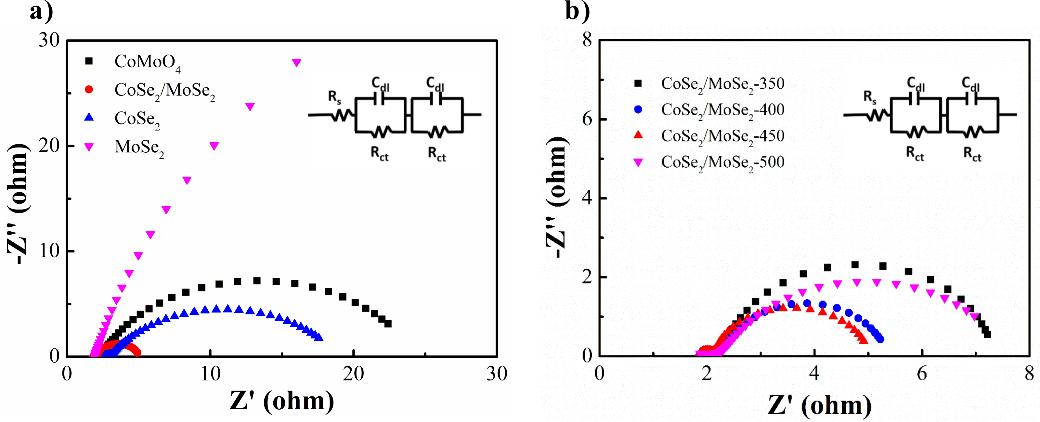


**Figure S5. (**a) Nyquist plots collected at 500 mV vs. SCE of CoMoO_4_, CoSe_2_/MoSe_2_, CoSe_2_, MoSe_2_; **(**b) Nyquist plots collected at 500 mV vs. SCE of CoSe_2_/MoSe_2_ with different temperatures.


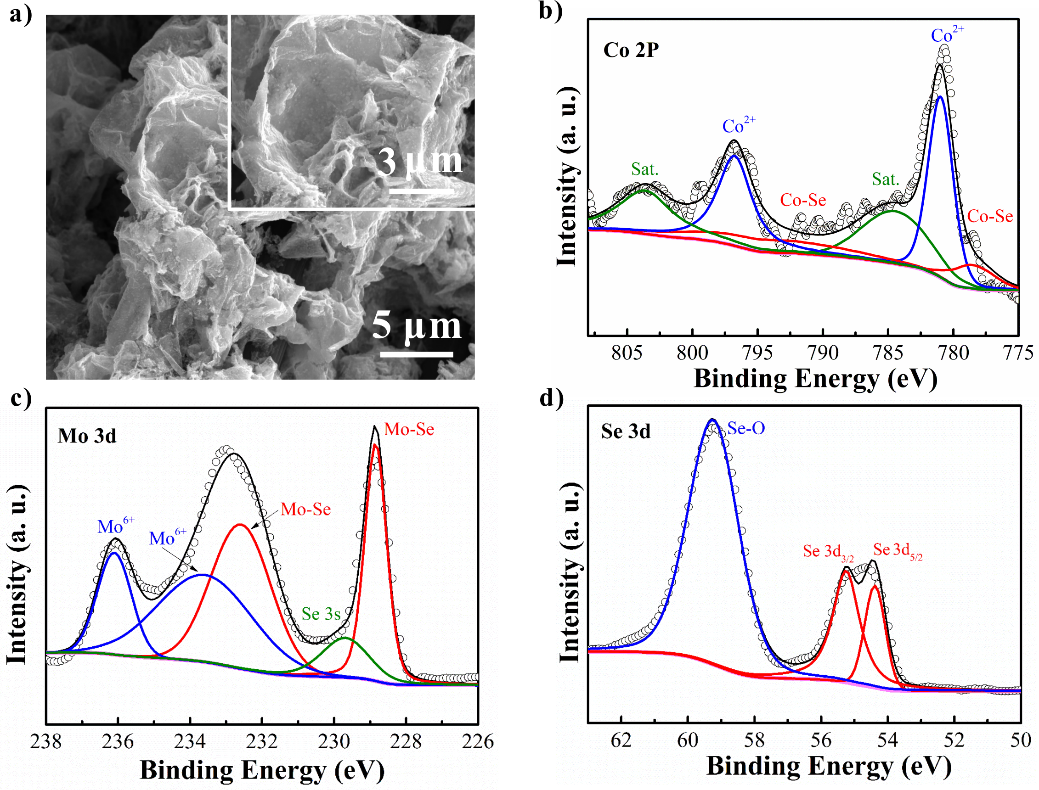


**Figure S6.** CoSe_2_/MoSe_2_ after galvanostatic cycling in 1 M KOH (a) FE-SEM image; (b) Co 2p XPS spectrum; (c) Mo 3d XPS spectrum; (d) Se 3d XPS spectrum.

**Table S1.** Comparison of hydrogen evolution reaction performance of several recently reported non-noble electrocatalysts in the alkaline medium.

| Catalyst | Electrolyte | η_10_ (mV vs.RHE) | Reference |
| --- | --- | --- | --- |
| CoSe_2_/MoSe_2_/CC | 1M KOH | 90 | **This work** |
| NiSe NWs/Ni Foam | 1M KOH | 96 | Angew. Chem. Int. Ed., 54 (2015) 9351-9355. |
| EG/cobalt selenide/NiFe-LDH | 1M KOH | 260 | Energy Environ. Sci., 9 (2016) 478-483. |
| o-CoSe_2_/P | 1M KOH | 104 | Nature Communications, 9 (2018) 2533. |
| CoSe_2_ NCs | 1M KOH | 520 | ACS Appl. Mater. Interfaces, 8 (2016) 5327-5334. |
| Co_0.75_Ni_0.25_Se/NF | 1M KOH | 106 | Nanoscale, 11 (2019) 7959-7966. |
| 1T MoSe_2_/NiSe | 1M KOH | 120 | Small Methods, 3 (2019) 1800317. |
| SWCNTs/MoSe_2_ | 1M KOH | 219 | ACS Nano, 13 (2019) 3162-3176. |

**Table S2.**  Comparison of oxygen evolution reaction performance of several recently reported non-noble electrocatalysts in the alkaline medium

| Catalyst | Electrolyte | η_10_ (mV vs.RHE) | Reference |
| --- | --- | --- | --- |
| CoSe_2_/MoSe_2_/CC | 1M KOH | 280 | **This work** |
| Ag-CoSe_2_ | 1M KOH | 320 | Angew. Chem. Int. Ed., 56 (2017) 328-332. |
| CoSe_2_ NCs | 1M KOH | 430 | ACS Appl. Mater. Interfaces, 8 (2016) 5327-5334. |
| CoSe_2_/DETA | 1M KOH | 392 | ACS Appl. Mater. Interfaces, 9 (2017) 39312-39317. |
| NiCo_2_Se_4_ holey nanosheets | 1M KOH | 295 | ACS Nano, 11 (2017) 9550-9557. |
| NiSe-Ni_0.85_Se/CP | 1M KOH | 300 | Small, 14 (2018) 1800763. |
| SWCNTs/MoSe_2_ | 1M KOH | 295 | ACS Nano, 13 (2019) 3162-3176. |
| 1T/2H MoSe_2_ | 1M KOH | 397 | Electrochim. Acta, 326 (2019) 134976. |
| CoSe_2_@MoSe_2_ | 1M KOH | 309 | Nanoscale, 12 (2020) 326-335. |

**Table S3.**  Comparison of the overall water splitting performance of several recently reported non-noble electrocatalysts in the alkaline medium

| **Catalyst** | **Mass loading**  **(mg cm^-2^)** | **Electrolyte** | **Current density j (mA cm^-2^)** | **Voltage of overall water splitting (V)** | **Refs.** |
| --- | --- | --- | --- | --- | --- |
| CoSe_2_/MoSe_2_/CC | 4 | 1 M KOH | 10 | 1.63 | **This work** |
| (Ni,Co)_0.85_Se NSAs | 1.5 | 1 M KOH | 10 | 1.65 | J. Mater. Chem. A, 6 (2018) 7585-7591. |
| a-CoSe/Ti mesh | 3.8 | 1 M KOH | 10 | 1.65 | Chem. Commun., 51 (2015) 16683-16686. |
| CoO_x_-CoSe | 1.7 | 1 M KOH | 10 | 1.64 | J. Mater. Chem. A, 4 (2016) 10933-10939. |
| [Co_0.85_Se@NC](mailto:Co0.85Se@NC) | 0.4 | 1 M KOH | 10 | 1.76 | J. Mater. Chem. A, 5 (2017) 7001-7014. |
| CoB_2_/CoSe_2_ | 0.4 | 1 M KOH | 10 | 1.73 | ACS Appl. Mater. Interfaces, 9 (2017) 39312-39317. |
| NiSe_2_/Ni | 2.6 | 1 M KOH | 10 | 1.64 | ACS Sustainable Chem. Eng., 6 (2018) 2231-2239. |
| 1T/2H MoSe_2_/MXene | 3 | 1 M KOH | 10 | 1.64 | Electrochim. Acta, 326 (2019) 134976. |
| Ni_3_Se_2_/CF | 3 | 1 M KOH | 10 | 1.65 | Catal. Sci. Technol., 5 (2015) 4954-4958. |
